# Unveiling barriers to maternal mental health in Pakistan: knowledge, attitudes and stigmas toward postpartum depression in social support systems

**DOI:** 10.3389/fpubh.2025.1527847

**Published:** 2025-06-09

**Authors:** Jawaria Shahzad, Laiba Nazim, Laraib Hussain, Hurais Malik, Rozina Mustafa, Muhammad Usama Jamil, Muhammad Abdullah, Mah Zaib Masood Awaisi, Syed Hassan Ahmed, Abdul Rehman, Muhammad Hudaib, Manahil, Ahmad Umais Ahad, Khabab Abbasher Hussien Mohamed Ahmed

**Affiliations:** ^1^Fazaia Ruth Pfau Medical College, Karachi, Pakistan; ^2^Nishtar Medical University, Multan, Pakistan; ^3^CMH Lahore Medical College & Institute of Dentistry, Lahore, Pakistan; ^4^Dow University of Health Sciences, Karachi, Pakistan; ^5^Pakistan Institute of Medical Sciences, Islamabad, Pakistan; ^6^Faculty of Medicine, University of Khartoum, Khartoum, Sudan

**Keywords:** postpartum depression, mental health, social support system, knowledge, attitude, Pakistan, stigma

## Abstract

**Background:**

Postpartum depression (PPD) poses a significant mental health concern for mothers globally. Despite a substantial prevalence, PPD often goes undetected and untreated, particularly in low- and middle-income countries (LMICs) like Pakistan. This study assesses the knowledge and attitudes toward postpartum depression among the social support system of pregnant women in Pakistan.

**Methods:**

A cross-sectional study was conducted at three healthcare institutions in Pakistan, investigating the knowledge and attitudes surrounding PPD among the support system of gravidarum women. Participants included a diverse sample of 363 individuals, above 18 years forming the social support system for pregnant women. Data were collected through interviews using validated questionnaires, providing insights into their demographic profiles, knowledge, and attitudes toward PPD. IBM-SPSS version 26.0 was used for data analysis, employing statistical tests like Kruskal-Wallis, Mann–Whitney U, Spearman correlation to examine associations.

**Results:**

The sample (*N* = 363) was demographically diverse, with participants aged 18–60 years, predominantly urban-dwelling (83.5%), educational levels ranged from uneducated (19.6%) to higher education (38%). Findings reveal a moderate level of awareness (44.6%) but a negative attitude (47.5%) toward PPD among participants. Stigmatizing concerns regarding disclosure highlights the need for non- judgmental support, while positive attitudes toward non-mystical explanations (Black Magic and Jinns) of PPD suggest progress in mental health understanding.

**Conclusion:**

This study highlights the need for targeted interventions to address the stigma surrounding PPD and promote supportive environments for pregnant women in Pakistan. Enhancing awareness and fostering open dialog about PPD within social support networks can play a crucial role in mitigating its impact on maternal mental health.

## 1 Introduction

Postpartum depression (PPD) is a prevalent and profound mental health concern experienced by mothers in the aftermath of childbirth (1). Characterized by a depressive episode typically occurring within the first year postpartum, PPD manifests with a myriad of symptoms, including diminished pleasure, anxiety, panic episodes, excessive crying, persistently low mood, weight fluctuations, fatigue, reduced self- esteem, and potentially suicidal ideation (2). Despite its prevalence, PPD often remains undetected and untreated, posing a significant challenge to maternal mental health (1). Globally, PPD affects an estimated 10 to 15 percent of mothers (3), with an approximate 14 percent prevalence rate among women worldwide (4). The incidence of PPD varies across populations, cultures, and risk factors, contributing to regional disparities (1). Notably, low- and middle-income countries (LMICs) have witnessed a disproportionately higher incidence of perinatal depression compared to their high-income counterparts (5). Bangladesh, India, and Nepal, with prevalence rates ranging from 10 to 39% (6). These high rates are influenced by various factors, including socioeconomic disparities, cultural norms, and limited access to healthcare services. In countries like Bangladesh and Nepal, the absence of structured perinatal mental health services has compelled women to depend largely on informal support systems, including family members and community networks (7).

In Pakistan access to professional assistance for maternal depression is limited, particularly in rural and low-income urban areas. While tertiary hospitals in urban centers offer psychiatric consultations, the integration of perinatal mental health services into primary healthcare remains inadequate due to significant barriers such as stigma, lack of trained personnel, and underfunding hinder widespread access to care, thus, PPD incidence has surged to alarming levels, reaching as high as 63.3% (8). Some initiatives, like the Lady Health Worker program, have attempted to incorporate mental health interventions at the community level; however, these efforts are not uniformly implemented across the country. Moreover, cultural perceptions and stigma surrounding mental health issues often prevent women from seeking the help they need (9). Despite these challenges, there have been promising developments, such as the “Happy Mother, Healthy Baby” program, which utilizes non-specialist providers to deliver cognitive-behavioral therapy-based interventions, demonstrating feasibility and acceptability in the Pakistani context (10).

Comprehensive systematic reviews across diverse income settings have revealed a concerning inverse association between postnatal depression and child health outcomes (5). PPD’s repercussions extend beyond the maternal sphere, potentially resulting in chronic or recurring depression, interpersonal difficulties, and potentially emotional, behavioral, and cognitive maldevelopment in the child (11). A constellation of risk factors has been identified, including a history of psychiatric illness, prenatal psychological distress, exposure to domestic violence, poor marital relationships, and insufficient social support (12). Previous research underscores the pivotal role of social support in postpartum practices (13). Research conducted in Pakistan underscores the pivotal influence of spousal and family support in mitigating PPD, highlighting a positive correlation between the absence of spousal support and PPD incidence (14). Consequently, robust perceived social support holds promise as an effective preventive measure that extends beyond the perinatal period (13). Despite the pervasive nature of PPD, there exists a glaring lack of awareness regarding this pressing issue. Women grappling with postnatal depressive or anxiety symptoms often confide in their social networks, emphasizing the pivotal role of family members in identifying and averting PPD. Conversely, the dearth of familial support emerges as a potent risk factor for PPD development.

Remarkably, Pakistan lacks surveys dedicated to examining community awareness, attitudes, and knowledge concerning postpartum depression. Existing research within the Pakistani context has predominantly centered on prevalence and predictors while overlooking the exploration of family members’ awareness. Consequently, a comprehensive investigation into the knowledge and attitudes of individuals responsible for the care of postpartum mothers, encompassing parents, siblings, friends, spouses, and in-laws, becomes imperative. This study endeavors to bridge a significant gap in the literature by conducting an in-depth investigation into the knowledge and attitudes surrounding postpartum depression (PPD) within the social support system of gravidarum women in the context of Pakistan.

## 2 Methodology

### 2.1 Study design and participants

This cross-sectional study was conducted at three prominent healthcare institutions in Pakistan, spanning from July 2023 to December 2023. Ethical approval was obtained from the institutional review and ethics boards of the respective institutions. The study population comprised individuals who formed the social support system for pregnant women, were above 18 years of age, and had provided care to women during their post-natal period at least once. Specifically, our participants included only family members—such as spouses, mothers-in-law, and siblings—who are traditionally the primary caregivers in the postpartum period in the Pakistani context. These individuals were selected given their significant influence on maternal mental health outcomes and their central role in shaping knowledge, attitudes, and stigma surrounding postpartum depression. Exclusions were made for individuals who were pregnant, had recently given birth, or had a history of postpartum depression (PPD) or other mental health conditions. The minimum sample size (*n* = 311) was calculated by the WHO sample size calculator 2.0, using one sample situation: 1.1 Estimate a population proportion with specified absolute precision, with 95% confidence interval, 0.05 margin of error, and 28% prevalence of PPD in the province of Sindh (15). Those meeting the inclusion–exclusion criteria were recruited for participation. Out of the 380 individuals approached 363 participants provided consent and were interviewed, resulting in a cooperation rate of 95%. No imputation methods were utilized, and all responses were included in the analysis.

Ethical approval for the study was obtained from Institutional Review Board FRPMC (Ref: FRPMC-IRB- 2023-12), Ethical Review Committee CMH Lahore Medical College & Institute of Dentistry (Ref: 614/ERC/CMH/LMC), and Institutional Ethical Review Board NMU Multan (Ref: 13131/NMU). The research adhered to ethical standards outlined in the Declaration of Helsinki of the World Medical Association for trials involving humans (16), as per the authors’ statements. Participants were provided with a consent form containing information about the study’s purpose, the voluntary nature of their participation, and the measures taken to protect their privacy and confidentiality. Participants were informed of their right to withdraw from the study at any point without consequences. To safeguard participant information, rigorous data security measures were implemented. All collected data were de-identified and securely stored on password-protected computers and servers. Personal information, such as names and contact details, was not linked to the collected data. Only the research team had access to the data, and any published results or reports did not contain information that could identify individual participants.

### 2.2 Questionnaire development and validation

The validated questionnaire utilized in this study was obtained with permission from the authors of the study (17), aligning with our research objectives and the constructs to be measured. Interviews were conducted by trained interviewers who received specific training to minimize interviewer bias. Despite the questionnaire being a self-administered instrument, the decision to conduct interviews was made to enhance the quality of data collection and ensure clearer communication with participants (18). Given the potentially sensitive nature of the topic the use of interviews allowed trained interviewers to address any participant concerns, and ensure that the questions were understood as intended To ensure effective communication and minimize miscommunication, the questionnaire was translated into Urdu prior to its administration, and the translated questionnaire underwent a rigorous review for relevance, clarity, and comprehensiveness. Thus to establish content validity, the questionnaire underwent a structured expert review process involving professionals from the fields of psychology, and psychiatry. These subject matter experts critically assessed each item for its conceptual relevance, and comprehensiveness in capturing the intended constructs. Informed by their evaluative feedback, several modifications were implemented—ambiguous or imprecise items were reformulated for enhanced clarity, and structural adjustments were made. These revisions collectively ensured that the finalized instrument was contextually appropriate, methodologically robust, and optimally aligned with the characteristics of the target population.

A pilot test was conducted with a sample of 20 individuals to identify potential issues, such as confusing questions, response options, or formatting problems. Feedback from pilot test participants was collected and used to refine and enhance the questionnaire. All identified flaws were addressed before the actual research study to ensure accuracy and reliability. Internal consistency was assessed through Cronbach’s Alpha score of 0.62 to evaluate the reliability of the questionnaire.

### 2.3 Measures

The questionnaire used in the study consisted of three sections.

#### 2.3.1 Demographic information

The first section collected demographic information from participants, including age, gender, monthly income, residence, educational status, marital status, parity, monthly income, and whether they were health professionals or not. Additional participants were asked about the recognition of the term PPD.

#### 2.3.2 Knowledge assessment

The second section assessed the knowledge of social support system members regarding PPD. The Knowledge about Postpartum Depression Questionnaire (KPPD-Q) comprises 15 items, including questions about prevalence, symptoms, risk factors, consequences, and treatments for PPD. With responses recorded as either “True,” “False” or “I do not know,” each correct answer was scored with one point. While incorrect and “I do not know” responses were scored as 0. The total knowledge score was calculated by summing the correct responses, resulting in a maximum score of 15. To facilitate interpretation, total scores were converted into percentage values (i.e., [participant’s score / 15] × 100) to reflect the proportion of correct knowledge. Higher percentages indicated greater knowledge of PPD. A knowledge score < 50% (score ≤ 7/15) was considered indicative of poor understanding, 50–69% (score 8–10/15) as moderate Knowledge, and ≥ 70% (score ≥ 11/15) as good knowledge.

#### 2.3.3 Attitude assessment

The third section focused on the attitudes of social support system members toward PPD. The attitudes about Postpartum Depression Questionnaire (APPD-Q) includes 17 items aimed at assessing an individual’s opinions and beliefs about PPD. Additionally, three additional questions addressing beliefs in black magic and supernatural beings (Jinns), as well as the potential humiliation associated with disclosing PPD, were included in the attitude section. These questions were designed with consideration for the beliefs and socio-cultural background of Pakistan, recognizing their profound influence on shaping attitudes at a broader level. Responses were rated on a 7-point Likert scale (ranging from 1 point for “strongly agree” to 7 points for “strongly disagree,” with 4 treated as the neutral midpoint.), with total scores ranging from 20 to 140 points (higher scores indicating negative attitudes). For negatively worded items (e.g., “Women should be strong enough to deal with PPD without treatment”), scoring was reversed. Thus, for all the items scores of 1.00–3.99 represented a positive attitude, 4.00 indicated neutrality, and 4.01–7.00 signified a negative stance. This interpretive framework allowed for nuanced analysis of specific areas of knowledge deficits and attitudinal stigma related to postpartum depression.

### 2.4 Data analysis

Data analysis was conducted using IBM SPSS Statistics version 26.0 (IBM Corp., Armonk, NY, United States). The analysis process was structured in several stages to comprehensively evaluate participants’ knowledge, attitudes, and the influence of demographic variables on these outcomes.

#### 2.4.1 Descriptive statistics

Descriptive statistics were applied to summarize and present the participants’ sociodemographic characteristics, including age, gender, and other relevant factors. Categorical variables were reported as counts and percentages, allowing for a clear depiction of participant distribution across different demographic groups. For quantitative measures of knowledge and attitude, mean scores and standard deviations (SD) were calculated. These descriptive statistics offered insight into the central tendencies and variability within each score, providing an overview of general trends in knowledge and attitudes among participants.

#### 2.4.2 Inferential statistics

Given the potential non-normal distribution of the knowledge and attitude scores, non-parametric tests were employed to assess relationships between these scores and various demographic and general factors. Specifically, the Mann–Whitney U test was used for comparisons involving two independent groups (e.g., gender differences in knowledge or attitude scores). For comparisons involving more than two independent groups (e.g., age groups or education levels), the Kruskal-Wallis test was applied. Dwass-Steel-Critchlow-Fligner (DSCF) pairwise comparisons were applied as a post-hoc test to determine which specific groups differed significantly. These non- parametric methods ensured robustness by accommodating potential deviations from normality, enhancing the reliability of our findings across different demographic comparisons.

#### 2.4.3 Correlation analysis

To further explore relationships between the computed scores of knowledge and attitude with demographic variables, Spearman’s rank-order correlation was used. This non-parametric method was chosen for its suitability with ordinal data and non-linear relationships, as it does not require assumptions of normality. Spearman’s correlation coefficients provided insights into the strength and direction of associations, illustrating how changes in demographic factors, such as age or education level, corresponded with variations in knowledge and attitude scores.

For all statistical analyses, a significance level of 𝑝 < 0.05 was adopted. Results with *p*-values below this threshold were considered statistically significant, underscoring findings of particular relevance within the context of this study.

## 3 Results

### 3.1 Study participant’s sociodemographic profile

In our investigation of the knowledge and attitudes of postpartum depression within the social support system of pregnant females, we observed a diverse sample of 363 participants (refer to Table 1). The gender distribution was nearly even with diverse distribution of ages ranging from 18 to 60 years old. The majority of participants resided predominantly in urban areas (83.47%). When examining the educational background of our participants, we found a wide spectrum of qualifications, from uneducated (19.56%) to higher education (38.02%). In terms of marital status, the majority were married. Regarding income levels, more than half of the participants reported monthly earnings of less than Rs. 50,000 (52.07%). Employment status indicated that over half of the participants were employed. Intriguingly, nearly half of the participants have already heard about this condition.

**Table 1:** Correlational analysis of demographic variables across total knowledge and attitude scores.

Variables	Demographics		KPPD-Q		APPD-Q	
	n	%	Correlation coefficient	Sig. (2-tailed)	Correlation coefficient	Sig. (2-tailed)
Gender						
*Male*	170	46.8%	0.058	0.273	−0.014	0.797
*Female*	193	53.2%				
Age						
*18–27 Years*	108	29.8%	−0.099	0.06	0.243	<0.001
*28–40 Years*	132	36.4%				
*41–50 Years*	71	19.6%				
*51–60 Years*	52	14.3%				
Residence						
*Urban*	303	83.5%	−0.159	0.002	0.088	0.096
*Rural*	60	16.5%				
Educational status						
*Uneducated*	71	19.6%	0.431	<0.001	−0.302	<0.001
*Basic Education*	3	0.8%				
*Primary Education*	48	13.2%				
*Matriculation*	8	2.2%				
*Intermediate*	95	26.2%				
*Higher Education*	138	38.0%				
Marital status						
*Married*	265	73.0%	−0.007	0.894	−0.199	<0.001
*Single*	98	27.0%				
Do you have children?						
*Yes*	251	69.1%	0.002	0.974	−0.242	<0.001
*No*	112	30.9%				
Your monthly income						
*< Rs. 50 K*	189	52.1%	0.074	0.162	−0.035	0.5
*Rs.50 K–Rs. 200 K*	146	40.2%				
*> Rs. 200 K*	28	7.7%				
Professional status						
*Student*	49	13.5%	0.029	0.578	0.155	0.003
*Unemployed*	110	30.3%				
*Employed*	197	54.3%				
*Retired*	7	1.9%				
Health professional						
*Yes*	50	13.8%	−0.287	<0.001	0.286	<0.001
*No*	313	86.2%				
Have you ever heard about PPD?						
*Yes*	180	49.6%	−0.473	<0.001	0.291	<0.001
*No*	183	50.4%				
Total Attitude Score Mean (SD)	66.5 (12.7)	47.50%	−0.224	<0.001	1	–
Total Knowledge Score Mean (SD)	6.7 (3.5)	44.6%	1	–	−0.224	<0.001

### 3.2 Knowledge

The analysis of participants’ knowledge about postpartum depression, offers valuable insights into their understanding of this critical issue. Among the 15 questions posed, the findings reveal varying levels of comprehension of different aspects of postpartum depression (presented in Table 2). The mean of the Total Knowledge score for the entire sample was found to be 44.6% (Mean score: 6.7, SD: 3.45). Notably, participants demonstrated a moderate to strong grasp of certain aspects, such as recognizing symptoms, i.e., severe sadness and irritability, within a period of more than 15 days [Mean Score: 0.56 (56%), SD: 0.50] and the importance of psychological intervention [Mean Score: 0.74 (74%), SD: 0.43]. However, there were areas where knowledge levels appeared less robust, including the understanding of the role of hormonal changes in causing PPD [Mean Score: 0.16 (16%), SD: 0.37], the effectiveness of the supplements or vitamins in the treatment [Mean Score: 0.28 (28%), SD: 0.45] and the vital role of support system, i.e., family and friends [Mean Score: 0.13 (13%), SD: 0.34].

**Table 2:** Results of knowledge section.

Questions	Mean score	St. deviation
1. Severe sadness and irritability, within a period of more than 15 days, are symptoms of postpartum depression.	0.56	0.496
2. Women with symptoms of depression or anxiety during pregnancy are more likely to have postpartum depression	0.51	0.501
3. Only women who did not wish to become pregnant develop postpartum depression.	0.43	0.496
4. Postpartum depression is mainly caused by hormonal changes.	0.16	0.367
5. Postpartum depression usually does not affect women’s appetite or sleep	0.55	0.499
6. Women with post-partum depression can respond as well as other women to their baby’s need.	0.42	0.494
7. Women with post-partum depression find it more difficult to respond to the needs of their partner and their children.	0.67	0.471
8. Negative thoughts about a baby or the possibility of hurting him are frequent in post-partum depression.	0.40	0.492
9. Postpartum depression is the reason in which women think of suicide.	0.29	0.454
10. Treatment of post-partum depression requires professional help	0.71	0.453
11. The use of supplements & vitamins is an effective treatment for post-partum depression	0.28	0.450
12. General practitioner cannot help a woman with post-partum depression.	0.29	0.457
13. Psychological intervention is effective in the treatment of postpartum depression.	0.74	0.437
14. The support of family and friends is enough to overcome post-partum depression.	0.13	0.336
15. The prevalence of postpartum depression in Pakistan is 28 to 63%.	0.53	0.500

### 3.3 Attitude

**Figure 1:**
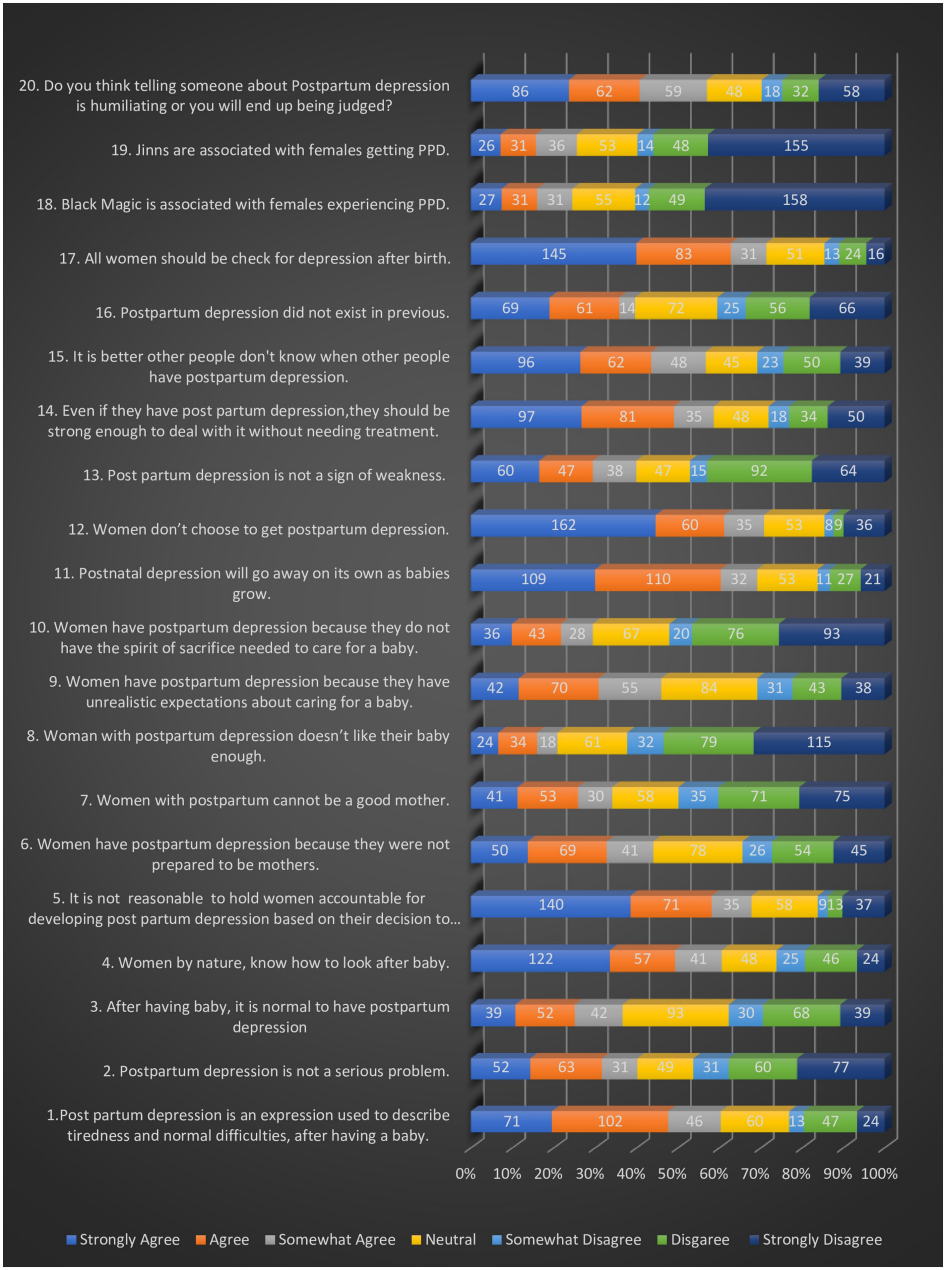
Distribution of responses across APPD-Q.

Furthermore, the study delved into the complex attitudes harbored within the social support system of pregnant women concerning Postpartum Depression (PPD), illuminating a diverse range of sentiments (see Table 3). The mean of the Total Attitude score for the entire sample was found to be 47.5% (Mean score: 66.5, SD: 12.7). Notably, respondents exhibited a multifaceted perspective on PPD (shown in Table 3; Figure 1), viewing it as an expression of typical postpartum challenges (Mean Score: 4.78, SD: 1.87). Simultaneously perceiving it as a relatively not-severe issue. While respondents mildly distanced themselves from the notion of holding women solely accountable for the development of PPD.

**Table 3:** Results of attitude section.

Questions	Mean score	St. deviation
1. Postpartum depression is an expression used to describe tiredness and normal difficulties, after having a baby.	4.78	1.886
2. Postpartum depression is not a serious problem.	3.81	2.168
3. After having baby, it is normal to have postpartum depression	3.94	1.860
4. Women by nature, know how to look after baby.	4.91	2.028
5. It is not reasonable to hold women accountable for developing postpartum depression based on their decision to have a baby.	2.76	1.975
6. Women have postpartum depression because they were not prepared to be mothers.	4.17	1.969
7. Women with postpartum cannot be a good mother.	3.61	2.068
8. Woman with postpartum depression does not like their baby enough.	2.96	1.939
9. Women have postpartum depression because they have unrealistic expectations about caring for a baby.	4.25	1.848
10. Women have postpartum depression because they do not have the spirit of sacrifice needed to care for a baby.	3.37	2.063
11. Postnatal depression will go away on its own as babies grow.	5.24	1.821
12. Women do not choose to get postpartum depression.	2.60	1.962
13. Postpartum depression is not a sign of weakness.	4.22	2.172
14. Even if they have postpartum depression, they should be strong enough to deal with it without needing treatment.	4.69	2.142
15. It is better other people do not know when other people have postpartum depression.	4.61	2.103
16. Postpartum depression did not exist in previous.	4.02	2.181
17. All women should be check for depression after birth.	2.56	1.796
18. Black Magic is associated with females experiencing PPD.	2.87	2.079
19. Jinns are associated with females getting PPD.	2.90	2.074
20. Do you think telling someone about Postpartum depression is humiliating or you will end up being judged?	4.51	2.138

Disturbingly, respondents held negative views believing that women should be strong enough to deal with PPD without needing treatment. (Mean = 4.69, SD = 2.41). Stigmatizing concerns surrounding humiliation and potential judgment in disclosing PPD were also present (Mean score = 4.51, SD = 2.14). Furthermore, the study unveiled substantial support for routine postpartum depression screening initiatives (Mean = 2.56, SD = 1.78). There were palpable slight positive attitudes toward cultural beliefs associating PPD with mystical phenomena such as black magic and jinns.

### 3.4 Sociodemographic influences on knowledge and attitude scores

In our pursuit of a deeper understanding of the interplay of sociodemographic factors on knowledge scores, several key findings emerged (see Tables 1, 4). Statistically significant differences were observed based on residence (*p* = 0.003), where individuals from urban areas had significantly higher scores than rural residents. Educational level showed multiple significant pairwise differences, particularly between uneducated individuals and those with intermediate (*p* < 0.001) and higher education (*p* < 0.001), and between intermediate and higher education groups (*p* = 0.006), indicating a positive association between education and knowledge. Similarly, those who had heard about PPD (*p* < 0.001) and those in a health profession (*p* < 0.001) demonstrated significantly greater knowledge scores than their counterparts. No significant differences were observed based on gender, age, marital status, or, monthly income.

**Table 4:** Association of demographic variables across total knowledge and attitude scores.

Variable	KPPD-Q		APPD-Q	
	Significant pairwise differences	*p*-value	Significant pairwise differences	*p*-value
Gender	*None*	0.273	*None*	0.796
Age	*None*	> 0.05	*28–40 > 18–27*	< 0.001
			*41–50 > 18–27*	< 0.01
			*51–60 > 18–27*	0.001
Residence	*Urban > Rural*	0.003	*None*	0.096
Marital Status	*None*	0.894	*Single > Married*	< 0.001
Education	*Intermediate > Uneducated*	< 0.001	*–*	–
	*Higher Education > Uneducated*	< 0.001	*Higher Education > Uneducated*	< 0.001
	*Higher Education > Intermediate*	0.006	*Higher Education > Intermediate*	0.004
Monthly Income	*None*	> 0.05	*None*	> 0.05
Have Children	*None*	> 0.05	*No > Yes*	< 0.001
Heard about PPD	*Yes > No*	< 0.001	*Yes > No*	< 0.001
Health Profession	*Yes > No*	< 0.001	*Yes > No*	< 0.001

Mann–Whitney U Test, Kruskal-Wallis Test & Dwass-Steel-Critchlow-Fligner pairwise comparisons Test applied.

Investigation into the association of demographic profiles with Attitude scores yielded several pivotal findings Significant differences were noted across age groups, with participants aged 18–27 scoring significantly lower than those aged 28–40 (*p* < 0.001), 41–50 (*p* < 0.01), and 51–60 (*p* = 0.001). Marital status also showed a significant effect, with single participants exhibiting significantly more positive attitudes compared to married individuals (*p* < 0.001). In terms of education, participants with higher education had significantly more positive attitudes than those who were uneducated (*p* < 0.001), and also more positive than those with intermediate education (*p* = 0.004). Additionally, individuals who had children (*p* < 0.001), had heard about PPD (*p* < 0.001), and worked in the health profession (*p* < 0.001) scored significantly higher on attitude compared to their counterparts. No significant differences were observed based on gender, income, or residence.

## 4 Discussion

Understanding what people know and how they feel about postpartum depression (PPD) among the support networks of pregnant women is crucial for providing meaningful help after childbirth. Our research set out to explore the understanding and emotions surrounding PPD within the support system of pregnant women. The study findings show that the average Total Knowledge score of 44.6% indicates a fair understanding among members of support networks regarding PPD. This suggests that while some individuals have a basic understanding of PPD symptoms and risk factors, there is room for improvement. Differences in knowledge levels between studies underscore the need for further investigation. Some studies report higher knowledge levels (2, 19), which could be influenced by various factors such as the study population, methods used, or cultural considerations. Factors like education, income, cultural beliefs, and societal attitudes toward mental health, as well as exposure to information about PPD, could contribute to differences in knowledge scores (20). Understanding the importance of social support in PPD recovery, informed families can recognize symptoms early, provide emotional support, and encourage seeking professional help (19). Therefore, targeted educational efforts within support networks are crucial to increase awareness about PPD, focusing on recognizing symptoms, understanding its prevalence and impact on maternal well-being, and knowing available support and treatment options (17).

Our Research reveals that the support circles surrounding expectant mothers are aware of the potential advantages of seeking help from mental health professionals to tackle PPD. Studies highlight that a significant proportion of participants acknowledge the advantages of psychological interventions offered by professionals in managing PPD (21). Thus suggesting that social support networks, including family members and friends, are aware of the importance of addressing PPD through appropriate interventions. This highlights the crucial role of integrating psychological assistance into prenatal care. Educating those within the support networks about the pivotal role professionals play in managing PPD can enhance their awareness and enable them to seek timely help when needed (22). A substantial body of research conducted in Western countries has established maternal postpartum depression (PPD) as a significant public health issue. PPD is defined as a non-psychotic depressive disorder of moderate severity that affects approximately 13% of women within the first 3 months following childbirth (12). In contrast, paternal perinatal depression has received comparatively less scholarly attention, despite growing recognition of its impact. The transition to fatherhood represents a major life change that can adversely affect men’s mental health. Studies have shown that paternal depression is associated with suboptimal parenting behaviors and reduced positive engagement in father–infant interactions (23). Moreover, emerging literature emphasizes that paternal perinatal depression (PPND) is also a significant concern, affecting up to 10% of fathers during the perinatal period (24).

The findings of this study shed light on how PPD is perceived by the participants. With an average Total Attitude score of 47.5%, where higher scores signify a more negative perception and lower scores indicate a more positive outlook, it becomes apparent that the overall attitude toward PPD tends to skew toward negativity among the individuals involved. Interestingly, the mean score hovers around the neutral point on the scale, suggesting a somewhat uncertain stance. This implies that while there may be some level of awareness or recognition of PPD, it does not necessarily translate into strongly positive or negative feelings. The prevailing cultural norms in Pakistan likely play a role in shaping these unfavorable attitudes toward PPD. The pervasive stigma surrounding mental health issues, including PPD, in various cultures often results in underreporting and reluctance to seek support (25). Limited awareness about PPD and its consequences may also affect attitudes. Studies indicate that there is a lack of understanding about mental health disorders in Pakistan, which leads to misunderstandings and negative views toward seeking help (26). Even though social support is essential for pregnant women, insufficient awareness and support for PPD could contribute to negative attitudes. The lack of empathy or understanding about PPD within the support network might hinder effective assistance (27). Variations in attitudes toward PPD among different populations in various regions or countries highlight the importance of considering cultural context and social factors when addressing mental health issues.

The negative viewpoints expressed by participants, suggesting that women should endure PPD without seeking assistance and perceive it as a minor issue, are worrying and highlight significant misconceptions about maternal mental health in this context. In Pakistan, mental health issues are sometimes seen as a sign of weakness, leading people to expect individuals to suffer silently. Traditional gender roles in Pakistan may also influence attitudes toward mental health, with women often expected to prioritize caregiving duties and suppress their own emotional needs (24). This societal perspective could further strengthen the belief that women should handle PPD on their own.

In Pakistan, the presence of stigma surrounding PPD creates a significant barrier for women seeking help and treatment. Cultural norms emphasize keeping mental health struggles private due to fears of judgment and societal stigma (28). Women may feel reluctant to seek assistance, fearing they will be perceived as weak or incapable if they disclose their experiences with PPD. To address this challenge, it is crucial to destigmatize PPD by fostering open discussions, increasing awareness about mental health, and providing support services that are easily accessible and culturally sensitive.

Moreover, our sample had a fair attitude toward viewpoints concerning cultural beliefs that link postpartum depression (PPD) with mystical events like black magic and jinns within the Pakistani community emphasizing the substantial influence of cultural factors on mental health perceptions. In Pakistan, deeply ingrained traditional beliefs regarding the supernatural causes of illnesses are prominent. Consequently, attributing PPD to mystical influences might serve as a coping strategy or an attempt to understand the condition within cultural contexts (29, 30). In societies where mental health problems are stigmatized, individuals may resort to mystical interpretations to avoid experiencing shame or facing social ostracism (31). These negative beliefs could negatively impact women’s health and well-being at a crucial time when they depend on the complete support of their families. Conversely, the positive attitudes toward explanations of PPD that do not involve mysticism suggest a growing recognition of mental health issues beyond traditional perspectives. This corresponds with studies emphasizing the importance of cultural factors in shaping attitudes toward PPD (32). Improving educational programs and awareness efforts might contribute to shifting attitudes toward mental health in Pakistan. Studies have indicated that higher levels of education are linked to a more accurate understanding of PPD (31).

Clinicians should be encouraged to provide comprehensive, culturally sensitive education on maternal mental health during both prenatal and postnatal care visits, ensuring that postpartum depression (PPD) is proactively addressed with patients and their families. This includes discussing symptoms, risk factors, and available treatment options, while emphasizing the crucial role of family support in early detection and intervention. From a policy perspective, these findings underscore the need for the development and implementation of national guidelines that mandate routine mental health screenings during the perinatal period, alongside targeted PPD awareness and education campaigns. Such policies should be integrated into existing maternal health programs, particularly in underserved and rural areas, to ensure equitable access to mental health resources. These strategies, when effectively implemented, can foster early identification, reduce stigma, and ultimately improve outcomes for mothers and their families across Pakistan. Given the increasing use of technology, digital interventions such as online educational platforms, mobile apps, or webinars could be integrated into prenatal care routines to increase awareness about PPD. These platforms can provide easily accessible information on recognizing symptoms, understanding the prevalence of PPD, and knowing available treatment options. This knowledge could be leveraged in telehealth services or online therapy platforms that allow for remote psychological counseling for women experiencing PPD (33).

However, our study has its limitations. The cross-sectional design hampers our ability to establish causality or infer temporal relationships among variables. While conducting interviews could mitigate some response bias associated with self-administered questionnaires, there is still a chance of interviewer bias influencing participant responses, despite our efforts to train interviewers. Moreover, the study’s restricted geographic scope might not fully capture the diverse perspectives of the broader Pakistani population. To address these challenges, future research could explore conducting interviews in a wider array of settings, including rural areas and community centers, to ensure a more inclusive sample. Involving participants with firsthand experiences of PPD could yield valuable insights into the attitudes of the social support system. Additionally, conducting interviews in multiple languages that reflect Pakistan’s linguistic diversity would promote inclusivity. Adopting longitudinal designs in future research to track attitude changes over time, along with diversifying sampling methods and integrating qualitative approaches, could provide deeper insights. Engaging community stakeholders in the research process and implementing educational initiatives to debunk myths about PPD and cultivate supportive attitudes within the community are also recommended.

## 5 Conclusion

This study reveals a moderate awareness (44.6%) of postpartum depression (PPD) among support system participants, contrasting with a negative attitude (47.5%) toward PPD. Stigmatizing concerns regarding disclosure highlight the need for non-judgmental support. Positive attitudes toward non-mystical explanations of PPD suggest progress in mental health understanding. To improve family cohesion and social support, targeted interventions should include culturally sensitive psychoeducation sessions for families, community-based support groups for spouses and extended family members, and integration of family counseling into routine maternal healthcare services. Targeted interventions can alleviate stigma and promote supportive environments for pregnant females facing PPD in Pakistan.

## Data Availability

The original contributions presented in the study are included in the article/supplementary material, further inquiries can be directed to the corresponding author.
